# Driving assistant using generative AI pre-generated messages in simulator-based driving assessment: A step towards low-cost simulator-based driving assessment

**DOI:** 10.1016/j.heliyon.2024.e35941

**Published:** 2024-08-12

**Authors:** Gunt Chanmas, Pittawat Taveekitworachai, Xiao You, Ruck Thawonmas, Chakarida Nukoolkit, Piyapat Dajpratham

**Affiliations:** a Graduate School of Information Science and Engineering, Ritsumeikan University, 2-150 Iwakura-cho, Ibaraki, 567-8570, Osaka, Japan; b College of Information Science and Engineering, Ritsumeikan University, 2-150 Iwakura-cho, Ibaraki, 567-8570, Osaka, Japan; c School of Information Technology, King Mongkut's University of Technology Thonburi, 126 Pracha Uthit Rd., Bang Mod, Thung Khru, 10140, Bangkok, Thailand; d Department of Rehabilitation Medicine, Faculty of Medicine Siriraj Hospital, Mahidol University, 2 Wanglang Road, Bangkoknoi, 10700, Bangkok, Thailand

**Keywords:** Driving assessment, Large language model, Vector database, CARLA driving simulator, LLMs-integrated system

## Abstract

This paper presents a novel approach for a low-cost simulator-based driving assessment system incorporating a speech-based assistant, using pre-generated messages from Generative AI to achieve real-time interaction during the assessment. Simulator-based assessment is a crucial apparatus in the research toolkit for various fields. Traditional assessment approaches, like on-road evaluation, though reliable, can be risky, costly, and inaccessible. Simulator-based assessment using stationary driving simulators offers a safer evaluation and can be tailored to specific needs. However, these simulators are often only available to research-focused institutions due to their cost. To address this issue, our study proposes a system with the aforementioned properties aiming to enhance drivers' situational awareness, and foster positive emotional states, i.e., high valence and medium arousal, while assessing participants to prevent subpar performers from proceeding to the next stages of assessment and/or rehabilitation. In addition, this study introduces the speech-based assistant which provides timely guidance adaptable to the ever-changing context of the driving environment and vehicle state. The study's preliminary outcomes reveal encouraging progress, highlighting improved driving performance and positive emotional states when participants are engaged with the assistant during the assessment.

## 1 Introduction

The ability to drive safely and confidently is crucial for maintaining independence and participating in various aspects of daily life [1], [2]. However, there are various groups of individuals who require driving assessment [3], [4], [5]. Driving assessment is an approach to determining the fitness-to-drive of individuals [6]. Such information is crucial for various purposes, e.g., granting driving permission [6], creating a personalized driving rehabilitation plan [7], and determining driving cessation [8].

Traditional driving assessment protocols involve, but are not limited to, on-road evaluations and stationed driving simulators. While on-road evaluations remain a reliable method for evaluating driving performance, this method is risky when driving on an actual road and costly in terms of financial and human resources [9]. Furthermore, access to this method can be limited [10]. Stationed driving simulators are introduced to overcome these concerns. These driving simulators offer a controlled environment for assessing driving skills, allowing for thorough evaluation [11]. Prior to the on-road evaluations, driving test-takers are usually recommended to undergo this method (pre-assessment) as it is considered safe and more time-and-cost effective compared to the on-road method. However, high-fidelity simulators are generally only available at research-focused institutions [12].

With technological advancements, there are some open-source driving simulators that closely resemble the real world and are able to run on commodity hardware, such as CARLA [13]. Adopting such low-computational-required simulators can mitigate financial burdens and open possibilities for exploring and integrating advanced technology including artificial intelligence. One such AI application is to integrate advanced driver assistance systems, intended to enhance driver capabilities related to perception, cognition, the selection of actions, and executing actions within a dynamic environment [14]. In addition, these simulators can have broad applications as an ante-pre driving assessment tool for determining whether to proceed with the traditional protocol.

The integration of natural language processing (NLP) enables human likeliness of in-vehicle driver notification [15]. Combining NLP with text-to-speech technology enables voice assistant which is considered an ideal modality to receive decision support without taking eyes off the road [16]. Past work suggested evidence showing that road safety is ultimately improved with the assistance of such notification providing road information [17], and the user acceptance rate has increased due to the enhancement of human-likeness in notifications. [18]. Implementing an ante-pre driving assessment could be beneficial in terms of minimizing financial concerns and broadening the accessibility of driving assessments. To the best of our knowledge, no study has implemented such ante-pre assessment in the existing driving assessment protocol.

In this work, we present a pioneering investigation that introduces an ante-pre assessment system, to enhance the assessment process and improve the precision of preliminary evaluations. With a recent breakthrough in Generative AI, this system leverages the power of a speech-based assistant with ChatGPT pre-generated messages to create a safe, cost-effective, and efficient assessment before going through the more costly traditional assessment protocol. By utilizing pre-generated messages, the proposed system achieves real-time interactions during driving in less than a second. Therefore, the proposed system mitigates ChatGPT's resource-intensive nature that can lead to slow responses [19], [20]. The speech-based assistant plays an important role in improving situation awareness and maintaining positive emotional states, specifically high valence and medium arousal [21]. The contributions of this study are to:

• Propose a low-cost-simulator-based driving assessment with an integrated speech-based assistant, powered by ChatGPT
• Explore the potential benefits of the speech-based assistant in providing guidance based on driving situations to enhance drivers' situation awareness in the driving assessment setting
• Investigate the role of the speech-based assistant in maintaining positive emotional states, specifically high valence and medium arousal, to mitigate frustration during driving in the driving assessment setting

## 2 Related work

This section aims to provide an overview of the use of simulator-based assessment techniques and innovative Large Language Model (LLM)-driven solutions, ultimately advancing the understanding in the domain of simulator-based driving evaluation. This section introduces relevant research endeavors centered around simulator-based driving assessment. Furthermore, it encompasses investigations that have delved into the integration of LLMs within driving applications, exploring their potential enhancements and contributions to the field.

### 2.1 Simulator-based driving assessment

Simulator-based driving assessment has emerged as a valuable tool for evaluating the driving abilities of individuals [22], [23], [24]. As traditional on-road assessments can be time-consuming, expensive, and potentially risky [9], researchers have explored the use of driving simulators to provide a controlled and safe environment for evaluating individuals' driving skills.

Previous reviews [25], [26] show that STISIM Drive© is the most popular choice among studies that utilized simulator-based assessment. STISIM Drive© is a stationary commercial driving simulator that offering a suite of features that many research institutes and rehabilitation centers find useful. These features include driving assessment features without relying on external software. Furthermore, these reviews also show that they employed other commercial driving simulators, e.g., Doron AMOS-2, AplusB Software, and Gridspace's GDS-300. The abundance of commercial driving simulators utilized across studies shows an important concern regarding impracticality in replicability and extensibility for institutes that cannot afford these kinds of simulators. Therefore, affordable alternatives are needed to make simulator-based driving assessment research more accessible. We aim to bridge this gap by introducing a driving assessment system enabling a preliminary evaluation before undergoing the assessment protocol.

### 2.2 LLMs in driving application

The application of LLMs in driving-related contexts has garnered increasing attention due to their potential to enhance various aspects of driving, including safety, navigation, communication, and user experience. LLMs, such as ChatGPT, have demonstrated remarkable capabilities in powerful zero-shot understanding and reasoning abilities in driving scenarios [27], making them valuable tools for addressing challenges in the field of driving. This related work section discusses areas where LLMs have been employed within driving applications.

A study by Du et al. [28] highlighted the benefits of ChatGPT toward driving applications as it can be integrated into an intelligent transportation system enabling the ability to provide traffic information. ChatGPT can additionally form human-vehicle interactions by assisting human drivers to perform high-concentration tasks including navigating through heavy traffic. Another study by Elhafsi et al. [29] introduced semantic anomaly detection of driving scenes with LLMs. With driving scenes simulated by CARLA, they extracted objects and integrated them into a prompt template to further classify unexpected situations. The prompt template is a predefined input format used to guide the generation of text and elicit specific responses from ChatGPT. The outcome illustrated the ability to identify semantic anomalies in complex scenarios of the LLM-based detection. We are inspired by the ideas presented in these studies to build a prompt template and tailor it to the need of the simulator-based driving assessment to elevate situation awareness and preserve positive emotional states, while assuring that driving subjects with subpar performance should not proceed to the next driving assessments. Furthermore, while LLMs provide a promising direction integrating into driving applications, slow response time remains a challenge to their use in real-time applications [19], [20].

## 3 Methodology

**Figure 1:**
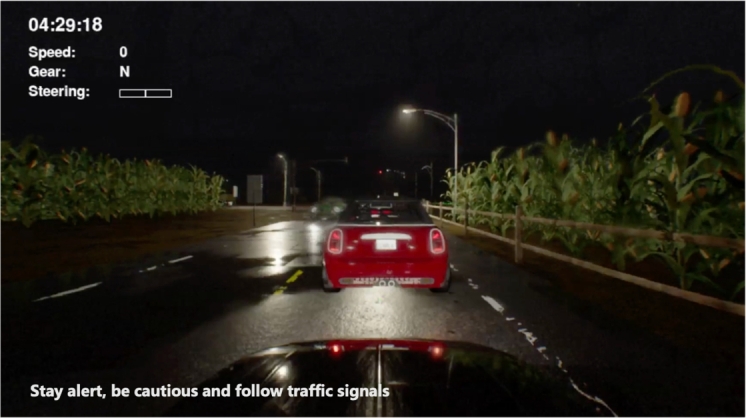
A screenshot of our proposed system; the top-left corner provides information including a time limit; the bottom-left shows the subtitle of the assistant's current voice message.

In the context of driving, LLMs can be employed to provide drivers with situation-aware and personalized advice to enhance their driving skills and ensure safe emotional states. By analyzing real-time data from the vehicle, road conditions, and the driver's actions, LLMs can offer insightful reasoning regarding driving environment awareness and other critical driving behaviors [27]. However, a limitation lies in the performance of LLMs which generally have long response times. To mitigate this concern, we propose a system, based on the CARLA driving simulator [13], consisting of two phases: (1) messages generation to generate text suggestions based on a given driving situation and vehicle state using ChatGPT and (2) real-time driving with an assistant to provide suggestions in a timely manner, or less than a second response time. The text suggestions are stored in a designated database prior to the experiment instead of entirely generating new suggestions during the experiment to achieve timely responses. Fig. 1 shows a screenshot of our system.

### 3.1 Proposed system: messages generation

**Figure 2:**
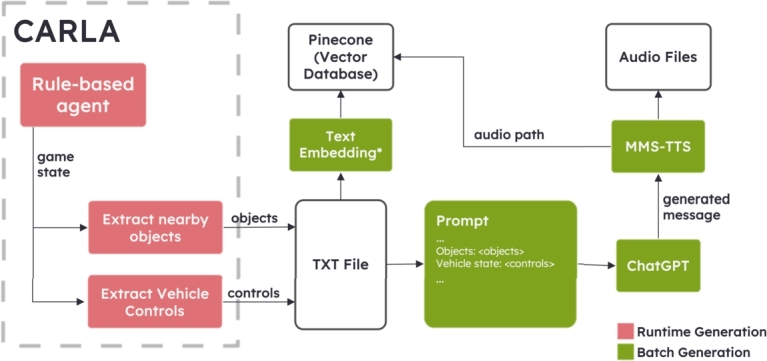
The first phase of our proposed system for messages generation; ^⁎^text embedding returns a 1,536-dimensional vector.

In the initial phase of the system, our focus is on implementing a data collection process for the driving environment. This involves setting up a comprehensive system to gather essential information from various driving scenarios and conditions. Once the data are successfully collected, the next step is to develop a message generation mechanism that could interpret the real-time driving environment data and generate appropriate messages according to the driving situation. These messages are vital as they form the core communication between the speech-based assistant and the system users (driving subjects in this context), providing crucial feedback and improving positive emotional states during their driving assessments. The overall system for the tasks in this sub-section consists of two phases, namely runtime data collection and batch message generation, and is available in Fig. 2.

**Figure 3:**
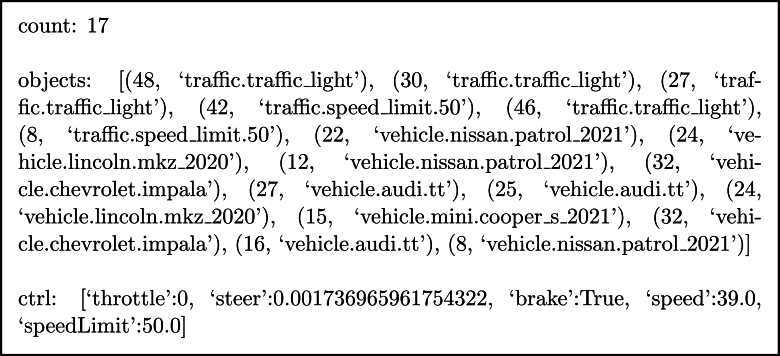
Example of collected data consisting of the total number of objects (count), the list of objects with its corresponding distance to the user's vehicle, and the vehicle control.

For the runtime data collection process, we utilize a rule-based agent provided by CARLA. This agent is employed to navigate through CARLA town 12 [30] as it provides diverse driving environments, simulating real-world driving scenarios using a pre-defined training route for a duration of approximately 20 hours. Throughout this extensive driving session, the agent diligently records information on nearby objects within 50 meters and the state of the agent, including speed, throttle, and steering, at its current location. The nearby objects involve only the simulated objects that have interacted with the simulator world, such as other vehicles that constantly move around the town, and traffic regulation objects that enforce changes in road conditions. These simulator elements are considered more important information compared to static objects including buildings and trees that have no interaction with the world. The data collected amounted to a total of 1,000 records, which were then saved into a structured text file for further analysis and processing as shown in Fig. 3. This set of data is available upon request.

**Figure 4:**
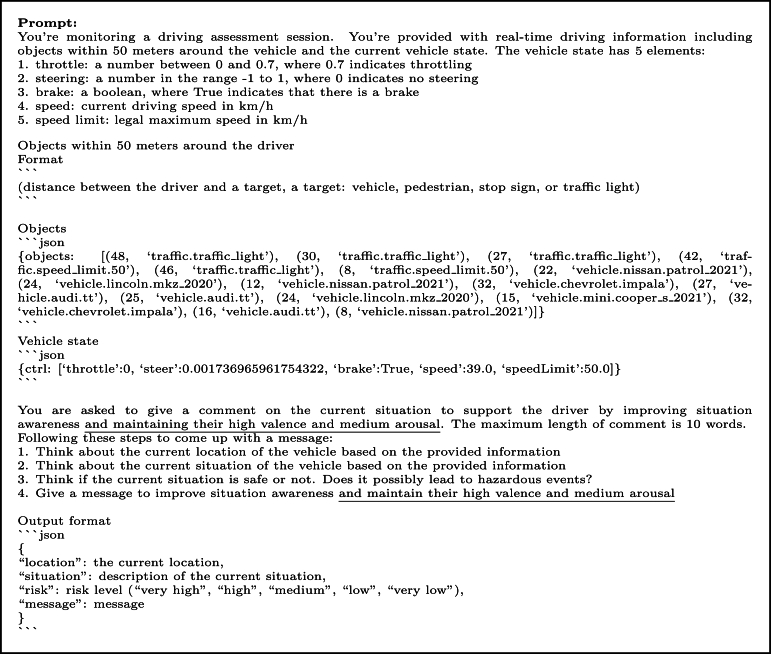
Example prompt, where the phrase for maintaining the driver's positive emotional state is underlined for illustration purposes.

**Figure 5:**
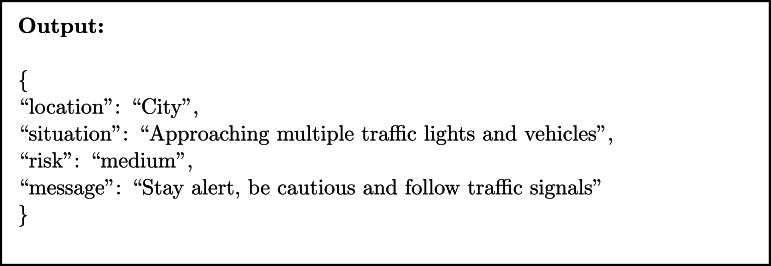
Example output.

For the batch message generation phase, we developed a Python Jupyter notebook that efficiently processes the collected data. This notebook is designed to carry out two important tasks. First, it reads and interprets the gathered data, including information about nearby objects and vehicle controls. The notebook incorporates a prompt designed to provide driving context and enhance positive emotional states as an input to ChatGPT and is outlined in Fig. 4 with the output shown in Fig. 5. In addition, we provide the distances calculated using the Euclidean distance between the driver and each object to determine the level of urgency as closer means more urgent. By combining the contextual information from the data with ChatGPT's abilities, our system could generate informative and contextually relevant messages to improve situation awareness and maintain safe emotional states.

In the next step of our process, we utilize MMS-TTS (Massively Multilingual Speech (MMS): Text-to-Speech Models) to generate speech audio files based on the generated text messages [31]. This enables us to transform the text messages into speech format and save them as files for later use. The nearby objects and vehicle controls are concatenated and then embedded using an efficient text embedding API provided by OpenAI [32]. This embedding process transformed the textual information into a high-dimensional vector representation, with the default number of 1,536 dimensions, capturing the essence of the combined data.

To manage and access these vectors effectively, we utilize the capabilities of Pinecone, a one-stop vector database solution [33]. The file paths, along with their corresponding embedded vectors, are stored as key-value pairs within the Pinecone vector database. This approach allows us to efficiently retrieve and access the relevant data during the real-time operation of our ante-pre assessment system with less than a second response time.

### 3.2 Proposed system: real-time driving with assistant

**Figure 6:**
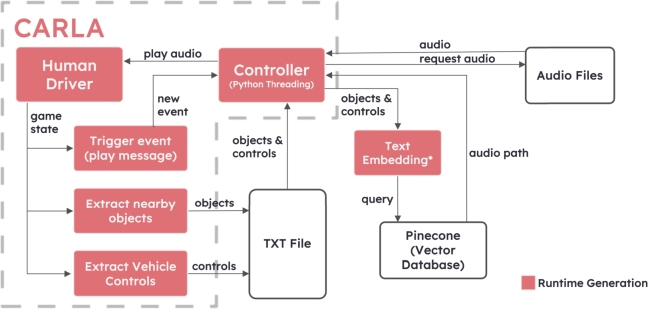
The second phase of our proposed system for real-time driving with an assistant; ^⁎^text embedding returns a 1,536-dimensional vector.

In this subsequent phase, we take a significant stride by integrating a speech assistant directly into the driving assessment system as shown in Fig. 6. To achieve this, we build a controller module by employing the Python threading package, allowing us to create a new thread dedicated to continuously monitoring changes in the surrounding objects during the driving session. As the user drives, the controller module remains actively engaged in analyzing the dynamic environment, ensuring timely and relevant feedback. Although, to date, there are no thresholds indicating which degree of the workload or distraction from a speech-based assistant exceeds an ordinary driving task [34], a 20-second cooldown of the assistant after assistance was provided is empirically added to mitigate this concern.

Similar to the process in Section 3.1, we follow the established approach of embedding the information about nearby objects and vehicle controls into a 1,536-dimensional vector using the aforementioned text embedding API. By combining this information, we generate a vector representation that encapsulates the real-time context of the driving situation. This vector is then utilized to query the Pinecone vector database efficiently based on vector similarity using cosine similarity.

**Figure 7:**
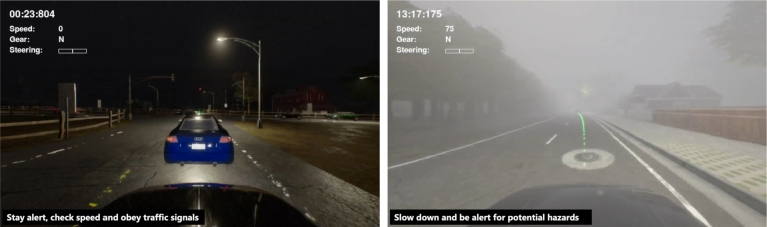
Screenshots of our system during driving with an integrated speech-based assistant. (Left) Feedback from the assistant when the driver arrived at a signalized intersection; (Right) Feedback from the assistant when the vehicle exceeded the speed limit of 70 km/h.

Upon retrieving the relevant information from the vector database, the speech assistant receives the corresponding audio path, which points to the pre-generated speech audio file. Subsequently, the speech assistant plays the appropriate audio feedback during the driving session, providing immediate and personalized responses to the user's driving performance while ensuring positive emotional states. Fig. 7 shows screenshots of the assessment with the assistant feedback in different situations. This integration of the speech assistant and the real-time monitoring of the environment allows for an interactive driving assessment experience. Users could now receive real-time guidance and support throughout their evaluation.

## 4 Experiments

**Figure 8:**
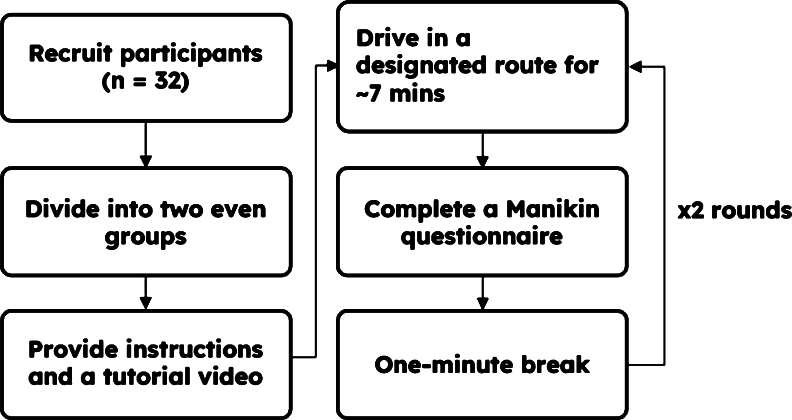
Flowchart of the experimental procedure.

Fig. 8 shows the experimental procedure that involved the recruitment of 32 participants for the driving assessment. They were healthy university students aged between 19 and 36 who, after the guidance session, agreed to participate in this preliminary study, which was conducted to investigate the efficiency of our system. These participants were then evenly divided into two groups, namely Group 1 (G1) and Group 2 (G2). Each participant underwent two rounds of driving in a designated route explained in Subsection 4.1, with each round lasting approximately 7 minutes where the driving performance, the details of which are given in Subsection 4.1, was measured. The experiment was conducted on a 17-inch laptop with RTX 3080 laptop GPU where users can navigate through the designated route using keyboard controls with W for acceleration, A or D for steering, and Space bar for brake. In the first round, G1 received assistance from the speech-based assistant during their driving sessions, whereas G2 performed the driving without the assistant.

To ensure our participants were adequately prepared for the assessment, detailed instructions and a tutorial video were provided before the commencement of the driving sessions. This allowed them to familiarize themselves with the driving simulator and the role of the speech-based assistant in providing real-time feedback during their driving sessions.

After completing each round, each participant was asked to complete a Manikin questionnaire [35] to assess their emotional states during the assessment. The questionnaire that was used in our study consists of 2 Likert scale 9-point questions: (a) valence where 1 and 9 indicate unhappiness and happiness, respectively, and (b) arousal where 1 determines calm and 9 shows excited emotion. Following the questionnaire, a one-minute break was provided to the participants before proceeding to the next driving round, where G1 and G2 drove without and with the assistant, respectively. By employing this experimental design, the study aimed to investigate the potential benefits of the speech-based assistant in enhancing drivers' situation awareness and emotional states during their driving sessions.

### 4.1 Driving route and evaluation

**Figure 9:**
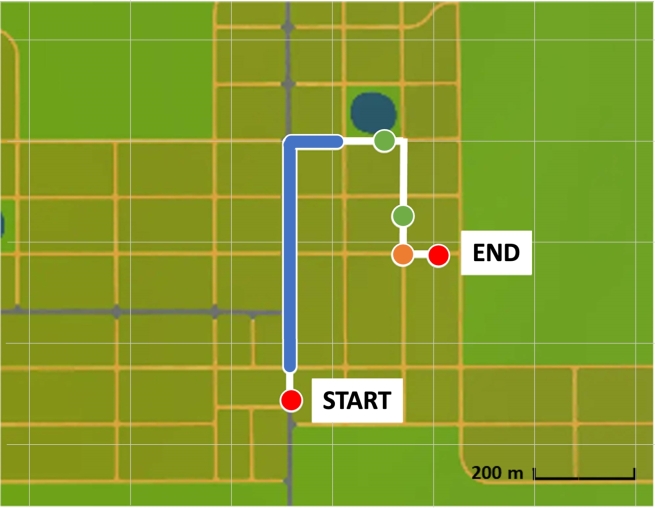
The overview of the selected route where a white line indicates the selected route; a blue shape represents one simple navigation scenario; green shapes represent two hazard perception scenarios; and an orange shape represents one trajectory planning scenario.

The selected driving route spans 1 kilometer and is estimated to take approximately 7 to 8 minutes to complete (see Fig. 9 for the overview of the route). The driving conditions for this route are set during the nighttime, adding an element of challenge and requiring heightened vigilance. The route has been carefully designed, adhering to an existing guideline that incorporates three types of driving scenarios [25]. These scenarios are composed of one simple-navigation-type scenario, which aims at assessing basic driving skills, two hazard-perception-type scenarios, to evaluate the ability to detect and respond to potential dangers, and one trajectory-planning-type scenario, testing the driver's capacity to plan and execute maneuvers effectively. The combination of these scenarios ensures a comprehensive assessment of the driver's abilities and situational awareness.

To measure the driver's performance during the driving evaluation on the selected route, several key indicators from CARLA are utilized. These indicators consist of (a) route completion (%), which quantifies the percentage of the route successfully completed by the driver; (b) the percentage of outside route (%) assessing the extent to which the driver deviates from the designated route; (c) the evaluation tracking the occurrences of neglecting stop signs and red lights, crucial factors that contribute to overall road safety; and (d) the evaluation monitoring the incidence of collisions involving pedestrians, vehicles, and objects. These indicators are recorded and analyzed using the CARLA leaderboard evaluation metrics (1) to identify potential areas for improvement and assess the driver's ability in navigating complex scenarios safely.

(1) $$DrivingScore=R\prod_{j}p_{j}^{n_{j}}$$

where *R* represents the percentage of the completed route deducted by the outside route percentage, $p_{j}$ is a fixed penalty factor of infraction *j* listed in Table 1, and $n_{j}$ denotes the occurrence number of infraction *j*; all numbers in Table 1 are default values from CARLA.

**Table 1:** List of all penalty factors based on infractions used in this study, where a small penalty factor indicates high severity.

Infraction (*j*)	Penalty factor (*p*)
Colliding with pedestrians	0.5
Colliding with other vehicles	0.6
Colliding with static elements	0.65
Running a stop sign	0.8
Running a red light	0.7
Scenario timeout (4 minutes)	0.7

### 4.2 Results and discussions

In this section, we perform statistical analysis—by employing the two-tailed paired *t*-test if the data follow a normal distribution; otherwise, the Wilcoxon signed-rank test—to evaluate the significance of differences in scores between various conditions and rounds. To assess the normality of the data distributions, we conduct Shapiro-Wilk tests and report the results in Table 2. By applying these tests, we can determine whether the differences are statistically significant, from which meaningful conclusions can be drawn about the impact of the speech-based assistant on participants' driving performance and emotional responses. Lastly, we analyze messages that were played during the experiment using BERTopic for topic clustering [36]. We choose BERTopic because it yields the most potential among embedding-based topic models when performing topic clustering with short texts [37].

**Table 2:** Results of Shapiro-Wilk Test, where the null hypothesis that the data are normally distributed is rejected if ^⋆^*p* < 0.05.

	*p*-value
Driving score — No Assistant	0.1409
Driving score — With Assistant	0.0113^⋆^

Total error — No Assistant	0.0049^⋆^
Total error — With Assistant	0.0016^⋆^

Valence — No Assistant	0.0019^⋆^
Valence — With Assistant	0.0278^⋆^

Arousal — No Assistant	0.0039^⋆^
Arousal — With Assistant	0.0925

#### 4.2.1 Driving performance and emotional states

Table 3 compares the driving score, total number of errors, valence, and arousal between driving with and without the speech-based assistant. The driving score and total error represent the overall driving performance of the participants. The results indicate that the use of the speech-based assistant during the driving assessments led to a significant improvement in participants' driving scores and a notable reduction in total errors. Specifically, the mean driving score increased from 33.70 (No Assistant) to 46.26 (Assistant), while the mean total error decreased from 3.94 (No Assistant) to 2.53 (Assistant). These changes, both with statistical significance, suggest a positive impact on participants' driving performance due to the assistant's presence, highlighting the effectiveness of our generated messages in conveying valid information about driving situations and their positive reception by participants in a timely manner.

**Table 3:** Mean and SD of driving score, total error, valence, and arousal comparison between no assistant and with assistant; ^⁎⁎^*p* < 0.01 and ^⁎⁎⁎^*p* < 0.001 vs No Assistant, while ^⋆^*p* < 0.05 vs the middle value of 5; those in bold represent the more preferable values.

	*n*	Average	SD	*p*-value	Statistical Test
Driving score — No Assistant	32	33.70	23.19	0.0050	Signed-rank test
Driving score — With Assistant	32	**46.26**^⁎⁎^	26.71		

Total error — No Assistant	32	3.94	2.82	0.0005	Signed-rank test
Total error — With Assistant	32	**2.53**^⁎⁎⁎^	1.98		

Valence — No Assistant	32	6.22	1.95	0.5413	Signed-rank test
Valence — With Assistant	32	**6.47**	1.54		

Arousal — No Assistant	32	6.06^⋆^	2.11	0.0155	Signed-rank test
Arousal — With Assistant	32	**5.41**	2.05	0.2698	*t*-test

Table 3 additionally provides insights into the valence comparison during the driving assessments with and without the speech-based assistant. To achieve safe emotional states, drivers should maintain positive valence [21], implying that the higher reported valence is considered better. The comparison of valence scores between driving sessions with and without the assistant reveals that participants reported slightly higher valence scores when the speech-based assistant was present during their driving assessments. Specifically, the mean valence score increased from 6.22 (No Assistant) to 6.47 (Assistant). Although no statistically significant difference was found, this indicates that the presence of the assistant during driving may have some impact on the driver's valence, as indicated by the findings.

Furthermore, both arousal levels of no assistant and with assistant are compared with the ideal middle value of 5, which indicates safe driving [21], [38]. Our findings revealed that when participants drove without the speech-based assistant, there was an average arousal score of 6.06 which is notably higher than the middle value of 5. Statistical significance is observed (*p* = 0.0155), suggesting that driving without the presence of the speech-based assistant potentially leads to unsafe driving. In contrast, driving with the assistant has a minimal change in an average arousal score of 5.41, which was not statistically significant (*p* = 0.2698). The result suggests that the assistant had a stabilizing effect on participants' arousal levels, helping to maintain them close to the ideal midpoint of 5 during the driving. This evidence suggests that the speech-based assistant plays a pivotal role in helping maintain emotional states consistent with safe driving.

#### 4.2.2 Message clustering

**Figure 10:**
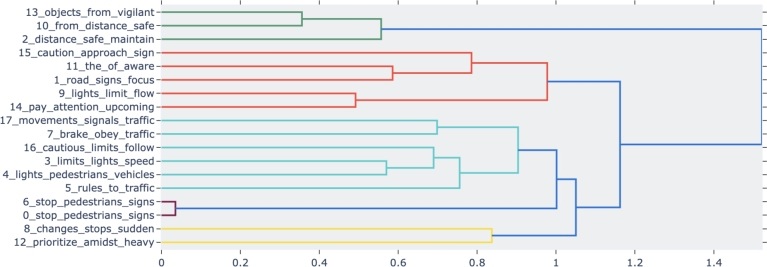
Results of topic clustering using BERTopic of all messages played during the experiment.

During the driving assessment, the speech-based assistant employs a cosine similarity algorithm to curate messages. In this session, we meticulously analyze the message logs of all 32 participants, resulting in a comprehensive dataset comprising 500 messages. The statistical summary of our message selection process reveals a mean distance of 0.98 and a standard deviation of 0.003 for the chosen messages. Subsequently, we perform a topic clustering with BERTopic and identify 5 distinct clusters, as visually represented in Fig. 10.

Starting from Cluster 1 (green), it primarily centers on the themes of vigilance and safe driving practices. Topics grouped within this cluster underscore the importance of maintaining awareness of nearby objects and adhering to safe following distances while driving. This cluster highlights the significance of staying alert to the surroundings and ensuring a safe distance from other vehicles or objects on the road, contributing to responsible and secure driving habits. Cluster 2 (orange) is focused on subjects concerning road signs, cautionary measures, and heightened awareness while driving. The cluster underscores the critical role of attentiveness to road signs, the need for a cautious driving approach, and the importance of being vigilant regarding upcoming road conditions and traffic signals. These themes collectively promote responsible and safety-conscious driving habits by encouraging drivers to stay informed and attentive to their surroundings on the road.

Cluster 3 (light blue) covers a diverse array of subjects related to traffic management, including adherence to traffic signals, compliance with traffic regulations, and the practice of cautious driving behaviors. It highlights the critical importance of respecting speed limits, adhering to traffic rules, and maintaining awareness of both pedestrians and vehicles while being attentive to traffic signals. Cluster 4 (purple) specifically addresses subjects related to pedestrian safety, particularly the act of stopping at pedestrian crosswalks and obeying stop signs within pedestrian zones. It underscores the significance of yielding to pedestrians and adhering to traffic regulations in areas designated for pedestrians, promoting safer interactions between drivers and pedestrians in these environments. Cluster 5 (yellow) is dedicated to topics related to adapting to abrupt alterations in driving circumstances and the importance of prioritizing actions when encountering heavy traffic or challenging situations. It offers practical strategies for effectively navigating unexpected stops and making informed driving decisions, especially in congested traffic scenarios.

## 5 Limitations

The participation of healthy university students in the current study offers valuable insights into the potential benefits of the speech-based assistant. Thereby, it is important to acknowledge the limitation of generalizing these findings to other groups of the population, who may require to adjust the computer setup. In addition, this study exclusively assesses driving performance using a single keyboard control configuration, potentially posing discomfort or challenges for individuals with limited or no prior familiarity with this specific setup. Lastly, the message generation mechanism of ChatGPT used in this study could operate as a black box, limiting transparency in understanding how messages are generated and the factors influencing their appropriateness, even with our instruction to provide information on its judgment.

## 6 Conclusions

In conclusion, this work presented a novel simulator-based driving assessment system with a speech-based assistant using ChatGPT pre-generated messages to achieve real-time interaction within less than a second with the driver. The primary aim of the system is to improve drivers' situation awareness and maintain positive emotional states during their driving assessments, which helps elicit safer driving behaviors. The study involved 32 participants divided into two groups evenly, subjected to driving sessions with and without the speech-based assistant, and the results demonstrated promising outcomes in improving driving performance among healthy participants.

The speech-based assistant proved instrumental in the driving assessment process, providing real-time feedback based on the current environment and vehicle state. This feedback contributed to enhanced situational awareness and positive emotional experiences for the drivers throughout the sessions. Statistical analysis revealed a significant difference in driving performance when utilizing the assistant, highlighting its potential to improve driving safety and performance. As a pioneering investigation in the integration of LLMs into driving assessment, the proposed ante-pre driving assessment can be implemented providing a cost-effective and accessible solution. We hold a positive outlook on employing such a system in ante-pre driving assessment for a wider population range as an effective way to gain preliminary insight before undergoing the traditional protocol.

## Ethics statement

This study involved an experiment lasting about 20 minutes, comprising a 2-minute introduction during which we provided necessary information about the experiment including driving rules and how to control the simulated vehicle. Subsequently, participants were tasked with driving the simulated vehicle in the driving simulator, following green dotted lines from start to finish. This route lasted about 7-8 minutes. After the driving session, participants were given a questionnaire to assess their emotional states. We then repeated the process with another driving session, also lasting approximately 7-8 minutes, followed by a follow-up questionnaire.

All participants were informed about the purpose of the research study, driving rules, and how to control the vehicle. Participation in this study was voluntary, and participants could decide to stop and leave the experiment at any time. They were informed that the information collected would be used as part of the study and would not affect them in any way. However, participants were not informed about the specific intervention, i.e., the speech-based assistant, utilized in this study. Randomization of the order across groups was employed to eliminate the effects of order. According to the guidelines of the authors' university, this study does not require ethical approval, and all human subject research procedures and protocols are exempt from review board approval.

Ritsumeikan University, King Mongkut's University of Technology Thonburi, and Mahidol University do not require approval for this kind of non-medical research. We consulted with the Research Ethics Review Committee Executive Office before conducting similar studies.

For your reference, please see other papers where the research was conducted at Ritsumeikan:

1. https://arxiv.org/ftp/arxiv/papers/2201/2201.10148.pdf They obtained informed consent, designed based on the research ethics guidelines at the authors' university, from all the participants.
2. https://ieeexplore.ieee.org/stamp/stamp.jsp?arnumber=9680732 The authors confirm that all human/animal subject research procedures and protocols are exempt from review board approval.
3. https://ieeexplore.ieee.org/stamp/stamp.jsp?tp=&arnumber=10017770

Research Ethics Handbook:

1. https://en.ritsumei.ac.jp/research/rosupport/starting-research/before/
2. https://drive.google.com/file/d/1Qysbjk6_3hWBGtvqYKPNbH2L205b7lw5/view?usp=sharing

## Funding

This work was supported in part by the 10.13039/501100005405Ritsumeikan University's Research Advancement Research Promotion Program for Acquiring Grants-in-Aid for Scientific Research.

## CRediT authorship contribution statement

**Gunt Chanmas:** Writing – original draft, Visualization, Software, Methodology, Investigation, Formal analysis, Data curation, Conceptualization. **Pittawat Taveekitworachai:** Writing – review & editing, Validation, Software, Methodology, Investigation, Data curation, Conceptualization. **Xiao You:** Investigation. **Ruck Thawonmas:** Writing – review & editing, Validation, Supervision, Resources, Project administration, Methodology, Formal analysis, Conceptualization. **Chakarida Nukoolkit:** Supervision, Methodology, Conceptualization. **Piyapat Dajpratham:** Supervision, Resources.

## Declaration of Competing Interest

The authors declare the following financial interests/personal relationships which may be considered as potential competing interests:

Ruck Thawonmas reports financial support was provided by 10.13039/501100005405Ritsumeikan University. Ruck Thawonmas also reports that he serves as an Associate Editor for the Computer Science Section of Heliyon. If there are other authors, they declare that they have no known competing financial interests or personal relationships that could have appeared to influence the work reported in this paper.

## Data Availability

Data will be made available upon request.

## Appendix A Supplementary material

MMC Manikin questionnaire. MMC
