# Supplementary material for: Driving assistant using generative AI pre-generated messages in simulator-based driving assessment: A step towards low-cost simulator-based driving assessment
Source: Heliyon. 2024 Aug 12;10(16):e35941. doi: 10.1016/j.heliyon.2024.e35941 (PMC11381592; doi:10.1016/j.heliyon.2024.e35941)
Supplement: MMC — Manikin questionnaire. [file mmc1.pdf]

Driving Simulator Questionnaire 2

pittawat2542@gmail.com Switch account

Not shared

* Indicates required question

Name *

Your answer

Group *

- G1
- G2

Manikin 1

Valence *

the emotional quality or positivity/negativity of an experience or emotion.

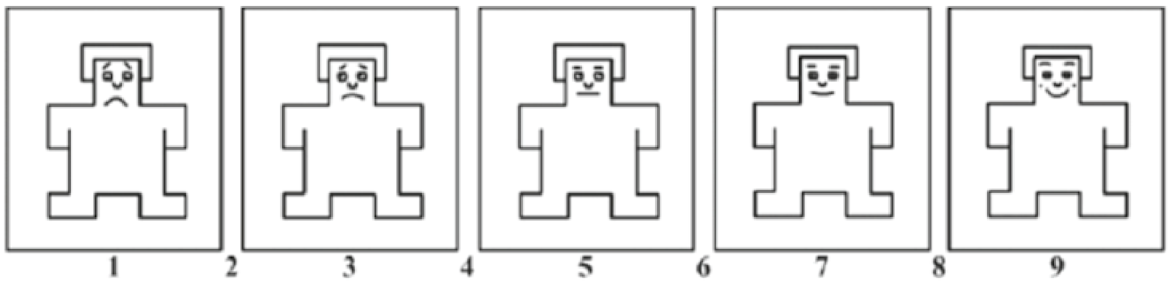

	1	2	3	4	5	6	7	8	9	
Unhappy	○	○	○	○	○	○	○	○	○	Happy

Request edit access

Arousal *

the level of physiological and psychological activation or energy associated with an experience or emotion.

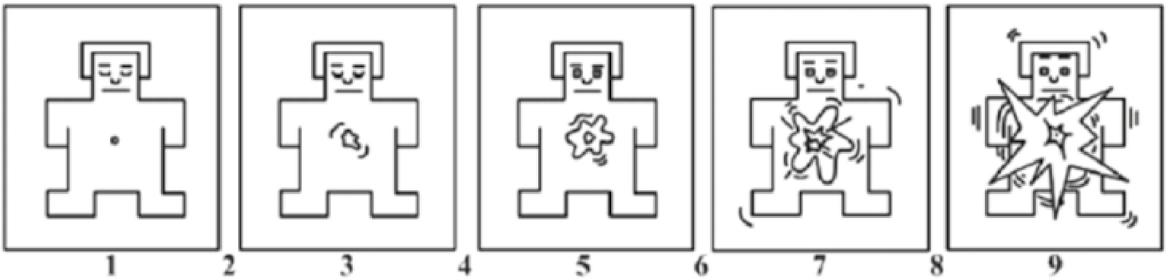

	1	2	3	4	5	6	7	8	9	
Calm	○	○	○	○	○	○	○	○	○	Excited

Dominance *

the degree of control or influence an emotion has over an individual's thoughts and behaviors

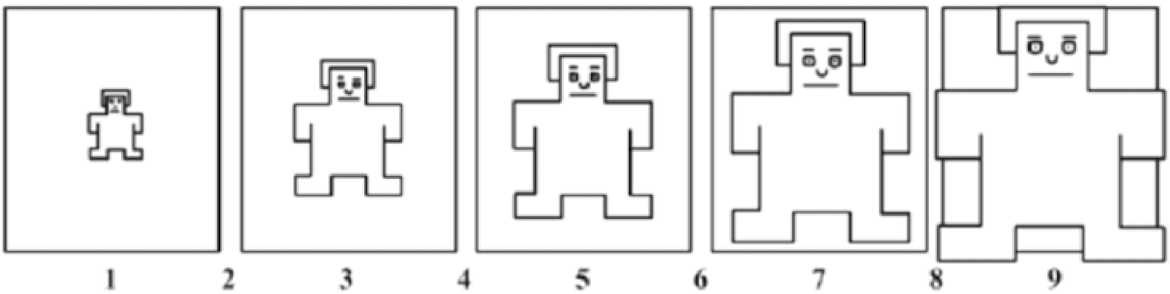

	1	2	3	4	5	6	7	8	9	
Controlled	○	○	○	○	○	○	○	○	○	Incontrol

Manikin 2

Request edit access

Valence *

the emotional quality or positivity/negativity of an experience or emotion.

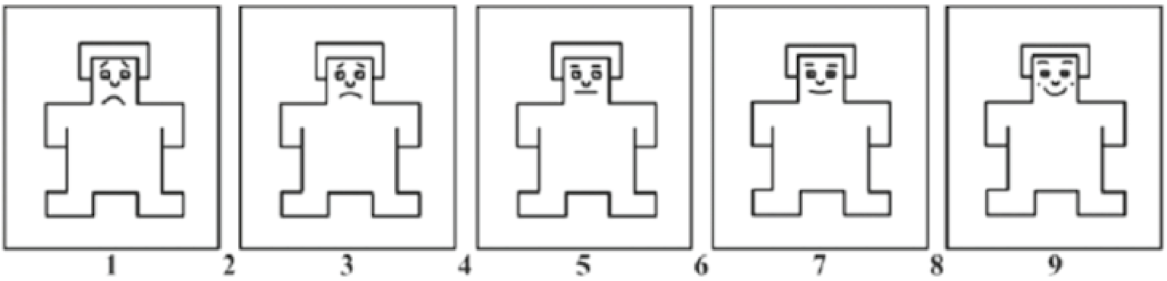

	1	2	3	4	5	6	7	8	9	
Unhappy	○	○	○	○	○	○	○	○	○	Happy

Arousal *

the level of physiological and psychological activation or energy associated with an experience or emotion.

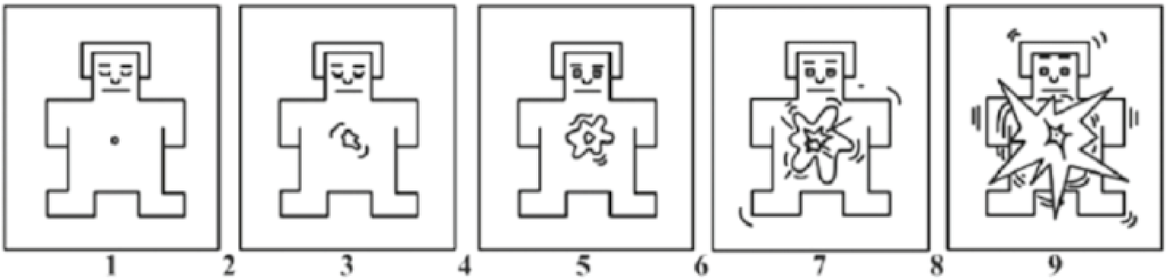

	1	2	3	4	5	6	7	8	9	
Calm	○	○	○	○	○	○	○	○	○	Excited

Request edit access

Dominance *

the degree of control or influence an emotion has over an individual's thoughts and behaviors

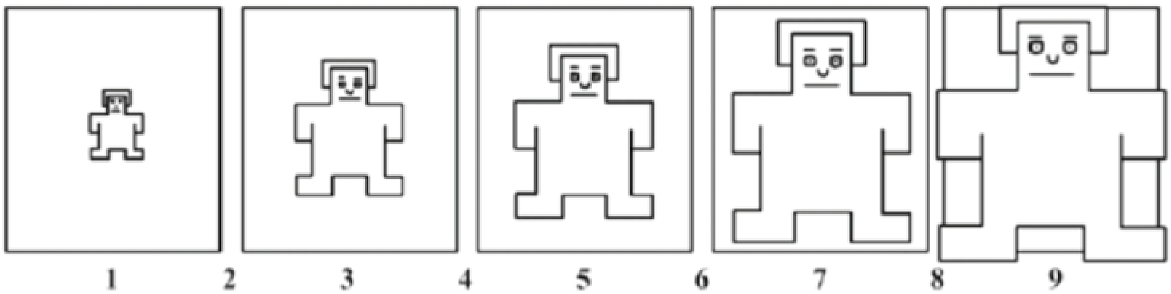

	1	2	3	4	5	6	7	8	9	
Controlled	○	○	○	○	○	○	○	○	○	Incontrol

Submit

Clear form

Never submit passwords through Google Forms.

This content is neither created nor endorsed by Google. Report Abuse - Terms of Service - Privacy Policy

Google Forms

Request edit access
